# Effects of dried distillers grains supplementation on intake, digestibility, metabolism, and performance of suckling beef calves on pasture

**DOI:** 10.1007/s11250-026-05177-5

**Published:** 2026-06-22

**Authors:** Edinael Rodrigues de Almeida, Johnnatan Castro Cabral Gonçalves, Jean Marcelo Albuquerque, Luanna Carla Coelho, Patrícia Siqueira Leite, Lilian Yukie Pacheco Toma, José Augusto Moura Godinho, Laura Ferrarez Ricardo, Julia Liliane Vieira, Cláudia Batista Sampaio, Edenio Detmann, Ériton Egídio Lisboa Valente, Sidnei Antônio Lopes

**Affiliations:** 1 Department of Animal Science, Federal University of Viçosa, Viçosa, MG 36570-900 Brazil; 2 Department of Agronomy, Federal University of Viçosa, Viçosa, MG 36570-900 Brazil; 3 Department of Animal Science, State University of Western Paraná, Toledo, 85960- 000 Brazil

**Keywords:** Animal development, Metabolizable protein, Tropical pastures, Cow-calf beef systems, *Creep-feeding*

## Abstract

The supply of metabolizable nutrients to suckling beef calves on pasture can improve their performance, increasing weaning weight and productivity in cow–calf systems. The objective was to evaluate the effects of dried distillers grains (DDG) inclusion on intake, digestibility, metabolism, and performance of suckling beef calves grazing tropical pastures. Forty-eight Nellore calves, 120 ± 35 days of age and 135 ± 23.4 kg of body weight (BW), were used. During 117 days, calves received one of the following treatments under a creep-feeding strategy: mineral mixture only, provided *ad libitum* (CONTROL); or concentrate supplementation (SUP, 6 g/kg BW, containing 225 g/kg crude protein) with 0% (SUP0), 48.4% (SUP48), or 96.8% (SUP96) DDG inclusion. Concentrate supplementation increased intake of dry matter (DM), crude protein (CP), non-fibrous carbohydrates (NFC), total digestible nutrients (TDN), and the CP/digestible organic matter ratio (*P* < 0.10). Digestibility of DM, CP, and NFC decreased linearly with increasing DDG inclusion. Serum glucose, insulin-like growth factor 1, and blood urea nitrogen concentrations were higher in calves on concentrate supplementation (*P* < 0.10). Concentrate supplementation increased average daily gain, weaning BW, and ribeye area (*P* < 0.10), with no differences related to DDG inclusion (*P* > 0.10). Supplementation improves beef calves nutritional characteristics, metabolism and performance. Additionally, DDG can be included in supplements for suckling beef calves grazing tropical pastures, with potential to replace traditional feedstuffs such as corn and soybean meal, without negatively affecting performance and serum metabolism.

## Introduction

In intensive systems of beef cattle production, calf supplementation is essential to obtain heavier animals, thereby enabling early slaughter of males and early breeding of females (Paulino et al. 2012; Lima et al. 2016). During the first weeks of life of beef calves in tropical systems under balanced forage supply, maternal milk is the main nutrient source, being sufficient to meet nutritional requirements for optimized gains close to 1 kg/animal/day. However, as lactation progresses, milk production decreases while the calf’s nutritional requirements increase, making milk insufficient to sustain this target from the 12th–14th week onward (Lopes et al. 2023).

Previous studies have demonstrated that creep-feeding supplementation of suckling calves effectively increases body weight at weaning (Valente et al. 2013; Lopes et al. 2016; Carvalho et al. 2019), as nutrients derived from grains are more digestible than those from pasture, particularly for calves whose fermentative capacity is still developing. Nevertheless, the adoption of supplementation may lead to increased production costs, potentially limiting the implementation of this strategy, since the cost of feed ingredients is a critical factor in strategic decision making. Therefore, the use of alternative feed ingredients may reduce supplementation costs and enhance the feasibility of its application in tropical beef production systems.

With the expansion of ethanol plants producing corn-based ethanol, the availability of dried distillers grains (DDG) have increased (Conab 2024). DDG are rich in digestible fiber and protein, particularly the rumen undegradable fraction (Buttrey et al. 2012). In addition to supplying nitrogen to ruminal microorganisms, supplementing grazing cattle with DDG is a strategy to increase the flow of amino acids to the intestine, thereby improving nitrogen use efficiency (Ferrari et al. 2021) and increasing metabolizable protein supply. Due to its nutritional value and relatively low cost, DDG has been widely used in Brazilian feedlot systems (Alhadas et al. 2023; Bremer et al. 2011; Depenbusch et al. 2009), primarily to replace feedstuffs with protein or energy profiles like traditional ingredients. However, there is a lack of studies evaluating its use in supplementation of grazing cattle, especially suckling calves.

We hypothesized that supplementation improve nutrient intake and digestibility, as well as metabolic and hormonal status, thereby enhancing the productive performance of suckling beef calves managed under a creep-feeding system. Additionally, we hypothesized that increasing inclusion levels of DDG, replacing soybean meal and ground corn while maintaining isonitrogenous supplements, would not impair performance or metabolic variables. Therefore, our objectives were evaluating the effects of supplementation and the effects of DDG levels in these supplements on forage and total intake, nutrient digestibility, serum metabolic and hormonal characteristics associated with the nutritional status, average daily gain and weaning weight of suckling beef calves grazing tropical pastures.

## Materials and methods

All procedures conducted during the experiment were approved by the Animal Ethics Committee for the Use of Production Animals at the Federal University of Viçosa, Brazil (CEUAP-UFV, protocol 011/2022). The study was carried out at the Beef Cattle Teaching, Research, and Extension Unit of the Department of Animal Science, Federal University of Viçosa, located in Viçosa, Minas Gerais, Brazil, from February to May 2023, covering both the rainy and the rainy-to-dry transition seasons.

A total of 48 suckling Nellore calves (30 males and 18 females), with an average age of 120 ± 35 days and an initial body weight (BW) of 135 ± 23.4 kg, born to multiparous cows (average 5 years of age and 480 kg BW), were used. Cow–calf pairs were randomly allocated to 12 paddocks of approximately 3.8 ha each, uniformly covered with *Urochloa decumbens*, and equipped with water troughs and covered feed bunks. Calves had restricted access to the feeding bunks through a creep-feeding system.

The experimental design was completely randomized, with four treatments, three replicates per treatment (paddocks/group as experimental units), and 12 observational units per treatment, ensuring balanced distribution of male and female calves within each group. The treatments consisted of mineral mixture only, provided *ad libitum* (CONTROL), or concentrate supplementation (SUP) containing 0% (SUP0), 48.4% (SUP48), or 96.8% (SUP96) dried distillers grains (DDG). This arrangement allowed the evaluation of both the effects of supplementation and the effects of increasing DDG inclusion, since DDG replaced soybean meal and ground corn in proportional amounts while maintaining isonitrogenous formulations. Supplements were formulated with 22.5% crude protein (CP), offered at proportion of 6 g/kg BW always at 11h00 a.m., with supplement allowance adjusted every 30 days according to intermediate BW measurements of the animals (Table 1).

**Table 1:** Ingredients composition of experimental supplements offered to suckling beef calves in creep feeding

Ingredient^2^	Treatment			
	CONTROL	SUP0	SUP48	SUP96
	*feed basis - g/kg*			
Ground Corn Grain	-	564	283	0
Soybean Meal	-	404	201	0
DDG	-	0	484	968
Pro-molasses	-	2	2	2
Mineral Mixture^1^	1000	30	30	30

^1^Percent composition: manganese sulfate 0.5; magnesium oxide 1.60; cobalt sulfate 0.05; copper sulfate 0.70; zinc sulfate 1.50; dicalcium phosphate 50.00; sodium selenite 0.09; potassium iodate 0.05; flower of sulfur 3.31 and sodium chloride 42.20. ^2^The calves were not supplemented (CONTROL) or supplemented without (SUP0) or with 48.4% (SUP48) or 96.8% (SUP96) inclusion of dried distillers grains (DDG) in supplements

The experimental period lasted 127 days, consisting of a 10-day adaptation phase to the supplements and paddocks, followed by 117 days of evaluation. During the adaptation phase, the dams were fed 100 g/animal/day of ground corn to stimulate bunk attendance and increase the time spent near the feed bunks, thereby promoting greater supplement intake by the calves. After this period, cows received only mineral mixture *ad libitum*.

### Experimental procedures and sampling

For forage characterization, samples were collected every 30 days to determine dry matter (DM) and potentially digestible dry matter (pdDM) per hectare. Each paddock was sampled at five random points using pruning shears and 0.5 × 0.5 m metal frames. For qualitative evaluation of the pasture, samples were obtained every 15 days through hand-plucking to simulate grazing. After collecting, forage samples were weighed and dried in a forced air oven at 55 °C for 72 h and subsequently ground in Wiley-type knife mills using 1- and 2-mm mesh screens.

To evaluate intake and digestibility, a 9-day trial was conducted. Fecal excretion was estimated using the external marker chromium oxide (Cr₂O₃), provided in paper cartridges at a dose of 10 g/calf/day. The marker was administered to all 48 calves through a metal probe, directly into the esophagus, always at 10h00 a.m. Titanium dioxide (TiO₂) was used to estimate individual supplement intake, incorporated into the supplement and supplied at 10 g/calf/day. The first five days of the trial were allocated to animal adaptation to the markers. From the sixth day onward, fecal samples were collected at different times (6h00 a.m., 10h00 a.m., 2h00 p.m., and 6h00 p.m.) to obtain representative samples from each animal (Sampaio et al. 2011). On the fifth day of the digestibility trial, a hand-plucked forage sample was collected from each paddock to evaluate the nutritive value of the forage consumed by the animals. Individual fecal samples (~ 200 g) were collected immediately after defecation, dried, and processed as described for forage samples. After grinding, a composite sample per animal was formed from the four collection days and used for chemical analyses. Indigestible neutral detergent fiber (iNDF) was used to estimate forage intake.

To estimate milk production of the dams, two collections were performed at 40 and 90 days after the start of the experiment. Calves were separated from the dams at 4h00 p.m. on the day before milk collection. At 6h00 p.m., calves were returned to their dams to allow suckling, aiming to completely empty the udder. Calves were then separated again at 7h00 p.m. and remained in a pen with access to water for a 12-hour period. The cows were allowed to graze in a nearby paddock, and on the following morning at 7h00 a.m., mechanical milking was performed after intravenous administration of 0.5 mL of oxytocin (10 IU/mL, Ocitovet^®^, Brazil) into the mammary vein. Daily milk yield for each cow was estimated as the production during the milking period (considering the time of calf separation and the time of milking) and adjusted to a 24-hour period (Lopes et al. 2022).

For performance evaluation and determination of shrunk average daily gain (ADG), animals were weighed after a 14-hour solid feed fast at the beginning and end of the experiment. Additionally, body condition score (BCS) of the dams, on a scale from 1 to 9 NRC (1996), was assessed by five trained technicians at the start and end of the trial. Ribeye area (REA) and subcutaneous fat thickness (SFT) were evaluated by ultrasound immediately after blood sampling. Ultrasound images were collected transversely across the *Longissimus dorsi* muscle between the 12th and 13th ribs and the rump. SFT was measured at the mid distal portion of the REA (LSFT). Additional images were obtained at the P8 site, measured at the intersection between the *Gluteus medius* and *Biceps femoris* muscles, located between the ischial and ilial tuberosities, where rump subcutaneous fat thickness (RSFT) was assessed. Ultrasound images were collected using an Aloka ultrasound machine (model SSD 500 V, Aloka Co., Ltd., Tokyo, Japan) with a 17.2 cm linear carcass transducer at 3.5 MHz, coupled with acoustic gel. Vegetable oil was applied as a coupling medium to ensure adequate contact between the probe and the animal’s body. Images for REA and SFT measurements were analyzed using BioSoft Toolbox^®^ II for Beef (Biotronics Inc., Ames, Iowa, USA).

### Chemical analysis

Forage, feces, and supplement samples were subjected to chemical analyses following the standard analytical procedures of the Brazilian National Institute of Science and Technology in Animal Science (INCT-CA) (Detmann et al. 2021). Samples ground to pass through a 1 mm sieve were analyzed for DM (INCT-CA method G-003/1), ash (INCT-CA method M-001/2), CP (INCT-CA method N-001/2), and ether extract (EE; INCT-CA method G-005/2). Neutral detergent fiber corrected for ash and protein (apNDF; INCT-CA method F-013/1) was determined using thermostable α-amylase without sodium sulfite, and insoluble protein in neutral detergent (NDIP; INCT-CA method N-004/2) was also quantified. iNDF of forage, feces, and supplement was estimated from samples ground to 2 mm and incubated in situ for 288 h using F57 bags (Ankom^®^) (INCT-CA method F-009/1). Additionally, fecal samples were analyzed for chromium (INCT-CA method M-005/2) and titanium (INCT-CA method M-007/2) contents. Organic matter (OM) was defined as 100 minus ash percentage.

Milk samples were analyzed for protein, fat, lactose, and total solids using infrared spectroscopy (Lactoscan SLP, Ultrasonic Milk Analyzer). Serum concentrations of total protein (biuret method, Bioclin^®^ K031), albumin (bromocresol green method, Bioclin^®^ K040), glucose (enzymatic colorimetric method, Bioclin^®^ K082), and urea (enzymatic colorimetric method, Bioclin^®^ K056) were quantified using an automated biochemical analyzer (Mindray BS200E, Shenzhen, China). Insulin-like growth factor type 1 (IGF-1) was measured using Siemens^®^ kits (Berlin, Germany) on an automated chemiluminescence analyzer at a commercial laboratory. Globulins were calculated as the difference between total protein and albumin. Serum urea nitrogen (SUN) was estimated as 46.67% of the urea concentration.

### Equations

Forage samples collected at ground level were used to estimate pdDM, according to the equation proposed by Paulino et al. (2008):

$$\:\mathrm{pdDM}\mathrm{=0.98\:x\:}\left(\mathrm{100-NDF}\right)\mathrm{+\:(NDF-}\mathrm{iNDF}\mathrm{)}$$

where: pdDM = potentially digestible dry matter (%); 0.98 = true digestibility of intracellular content; NDF = neutral detergent fiber (%); iNDF = indigestible neutral detergent fiber.

Fecal dry matter excretion was estimated as the ratio between the amount of chromium oxide administered and its concentration in the feces. Individual supplement intake (ISI) was calculated using the following equation:

$$\:\mathrm{ISI\:}\left(\mathrm{g/day}\right)\mathrm{=}\frac{\mathrm{FExIFc}}{\mathrm{IGs}}\mathrm{\:x\:}\mathrm{SupG}$$

where: ISI = individual supplement intake (g/day); FE = fecal excretion (g/day); IFc = indicator concentration in the animal’s feces (g/g); IGs = indicator provided in the supplement offered to the group (g/day); SupG = amount of supplement offered to the group of animals (g/day).

Individual forage dry matter intake (IFDMI) was estimated using the internal marker iNDF according to the equation proposed by Detmann et al. (2001):

$$\:\mathrm{IFDMI=}\frac{\mathrm{[}\left(\mathrm{FE\:x\:}\mathrm{iNDFfeces}\right)\mathrm{-(SDMI\:x\:}\mathrm{iNDFsup}\mathrm{)]}}{\mathrm{iNDFforage}}\text{}$$

where: IFDMI = individual forage dry matter intake (kg/day); FE = fecal excretion (kg/day); iNDFfeces = concentration of iNDF in the feces (kg/kg); SDMI = supplement dry matter intake (kg/day); iNDFsup = concentration of iNDF in the supplement (kg/kg); and iNDFforage = concentration of iNDF in the forage (kg/kg). Total dry matter intake (TDMI) was calculated as the sum of forage and supplement intake.

### Statistical analysis

The experiment was conducted using a completely randomized design with four treatments and four experimental units per treatment (i.e., groups of animals/paddock). The experiment was analyzed according to the following model:

$$Y\,ijkl = \mu + Ti + g\left( i \right)j + sk + \varepsilon ijkl$$

where: *Y*_*ijk*_ *=* represents the response measured in animal I, of sex *k*, belonging to group *j* and subjected to treatment *i*; µ = is the overall mean; T_i_ = is the fixed effect of treatment *i; g*_*(i)j*_ = is the random effect of the group of animals (i.e. paddock) *j* nested within treatment *i*, assumed to be NIID (0, σ²_i_/ⱼ); sₖ is the fixed effect of calf sex (included as a control factor); and *ε*_*ijk*_ = is the random error, unobservable and assumed to be NIID (0, σ_ɛ_^2^).

Treatment sum of squares were further decomposed into orthogonal contrasts to evaluate the overall effect of supplementation (CONTROL vs. SUP) and the linear and quadratic effects of DDG inclusion in the supplement. All statistical analyses were performed using the MIXED procedure of SAS (Statistical Analysis System, version 9.4), adopting a significance level of α = 0.10. A significance level of *P* < 0.10 was adopted due to the inherent variability of grazing conditions, aiming to reduce the probability of Type II error and improve the detection of biologically relevant treatment effects.

## Results

**Fig. 1:**
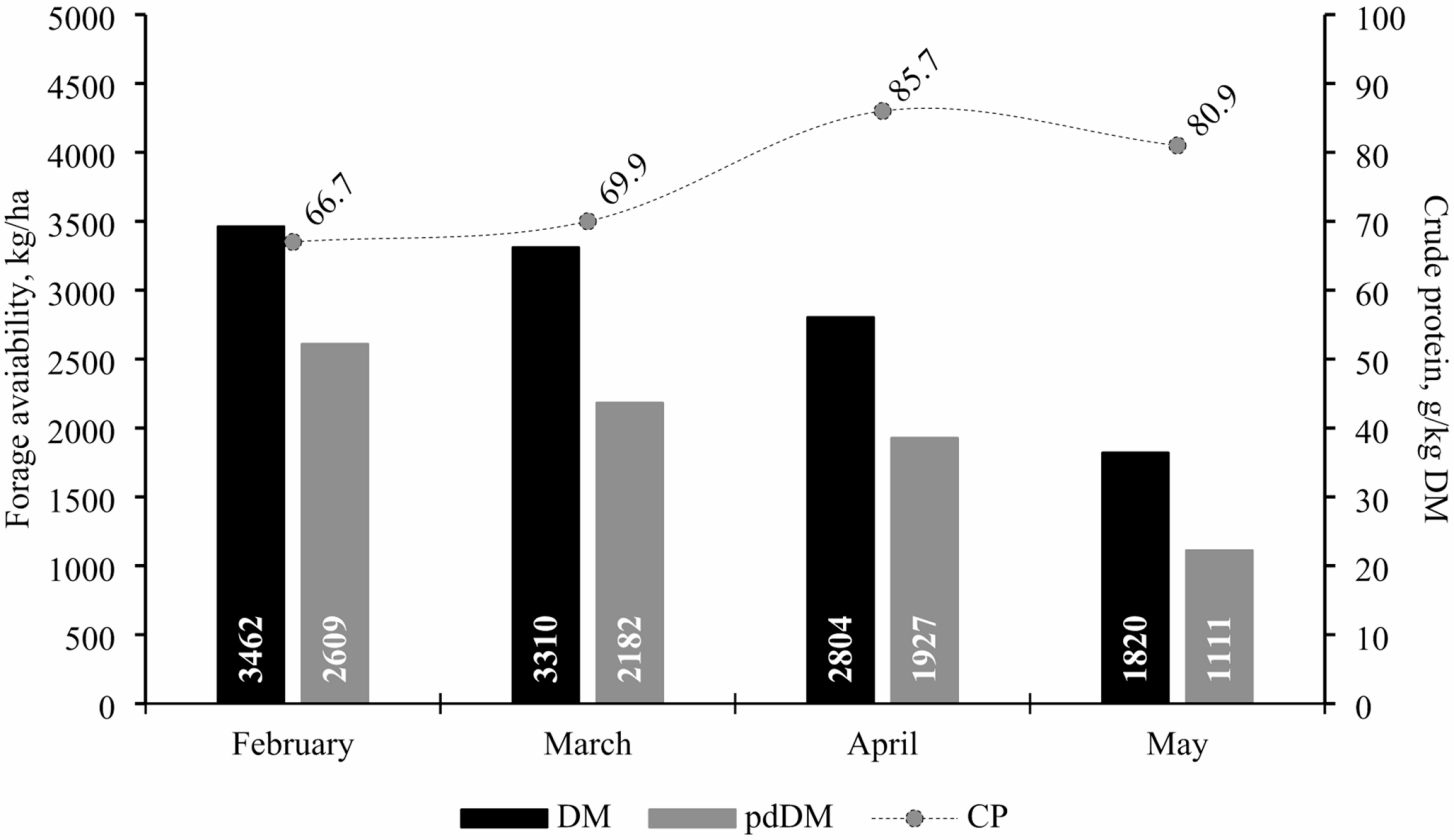
Monthly averages of dry matter (DM) availability, potentially digestible dry matter (pdDM, kg/ha), and crude protein (g/kg DM) of *Urochloa decumbens*, available to suckling beef calves (supplemented or non-supplemented) and their dams in a tropical creep-feeding system

The average availability of DM and pdDM during the experimental period was 2.85 and 1.96 t/ha, respectively, which resulted in an average availability of 174 g/kg and 117 g/kg BW (Fig. 1). The forage collected through simulated grazing had an average CP content of 75.7 g/kg DM (Table 2).

**Table 2:** Chemical composition of supplements and monthly chemical composition of *Urochloa decumbens* pastures, offered to suckling beef calves in a tropical creep-feeding system

Item^1^	Supplement^2^			Months^3^			
	SUP0	SUP48	SUP96	February	March	April	May
*g/kg DM*							
DM	891	904	913	281 ± 2.7	319 ± 19.5	324 ± 16.4	269 ± 28.6
OM	41.0	40.4	43.0	907 ± 4.2	908 ± 3.7	905 ± 4.5	904 ± 4.0
CP	224	224	224	66.7 ± 6.6	69.6 ± 10.6	85.7 ± 6.1	80.9 ± 15.3
EE	43.2	42.5	43.6	13.5 ± 0.2	13.5 ± 0.3	13.4 ± 0.7	14.0 ± 0.8
apNDF	147	333	537	658 ± 0.7	662 ± 1.1	648 ± 6.7	611 ± 8.8
NFC	510	341	146	169 ± 4.9	163 ± 11.1	158 ± 7.8	198 ± 12.8
iNDF	43.4	64.4	80.8	226 ± 1.1	275 ± 2.5	272 ± 15.9	303 ± 72.0
*g/kg CP* ^*4*^							
NDIP	27.6	52.3	74.3	122 ± 12.2	171 ± 22.7	167 ± 18.7	184 ± 33.0

^1^DM = dry matter; OM = organic matter; CP = crude protein; EE = ether extract; apNDF = neutral detergent fiber corrected for ash and protein; NFC = non fibrous carbohydrate; iNDF = indigestible neutral detergent fiber; NDIP = neutral detergent insoluble protein. ^2^Supplement with 0.0% (SUP0), 48.4% (SUP48) or 96.8% (SUP96) of dried distillers grains inclusion. ^3^Mean ± standard deviation of the mean. ^4^Expressed as total percentage of crude protein

Milk production and composition of the cows were not affected by calf supplementation (*P* > 0.10). On average, milk production was 6.3 kg/day (Table 3). This reflected the absence of a supplementation effect on milk dry matter intake by calves (*P* = 0.12) (Table 4).

**Table 3:** Milk yield and composition of Nellore dams nursing calves supplemented with different supplements during the suckling phase in a creep-feeding system

Item^1^	Treatment^2^				SEM^3^	*P*-value^4^		
	CONTROL	SUP0	SUP48	SUP96		CONTROL × SUP	L	Q
	*kg/day*							
Milk yield	6.01	6.48	6.27	6.52	0.386	0.38	0.93	0.63
FCM	7.02	7.55	7.59	7.34	0.390	0.32	0.71	0.77
	*g/kg*							
Protein	33.4	33.4	33.2	34.0	0.28	0.60	0.16	0.23
Fat	52.9	51.1	54.2	49.3	1.91	0.56	0.52	0.12
Lactose	50.1	50.1	49.9	51.1	0.42	0.60	0.16	0.22
Total solids	146	143	146	143	1.6	0.54	0.90	0.16

^1^Milk yield; FCM = 4% fat corrected milk yield. ^2^Dam’s calves were not supplemented (CONTROL) or supplemented without (SUP0) or with 48.4% (SUP48) or 96.8% (SUP96) inclusion of dried distillers grains, in a tropical creep-feeding system. ^3^SEM: Standard error of the mean. ^4^CONTROL × SUP, contrast between supplemented and non-supplemented calves. L and Q = linear and quadratic effects of treatments. Significative when *P* < 0.10

**Table 4:** Voluntary intake of beef calves that received different supplements during suckling phase in a tropical creep-feeding system

Item^1^	Treatment^2^				SEM^3^	*P*-value^4^		
	CONTROL	SUP0	SUP48	SUP96		CONTROL × SUP	L	Q
	*kg/day*							
DM	2.41	3.31	3.13	2.96	0.101	< 0.001	0.043	0.99
DMF	1.47	1.23	1.11	0.94	0.141	0.051	0.19	0.88
DMS	-	1.13	1.02	1.01	0.102	-	0.45	0.72
DMM	0.88	0.98	0.96	0.99	0.047	0.12	0.85	0.69
OM	2.22	3.07	2.91	2.75	0.091	< 0.001	0.042	0.97
CP	0.35	0.56	0.54	0.54	0.008	< 0.001	0.15	0.85
EE	0.34	0.40	0.41	0.40	0.022	0.036	0.94	0.69
apNDF	0.94	0.96	1.06	1.15	0.076	0.22	0.11	0.95
NFC	0.25	0.80	0.53	0.30	0.040	< 0.001	< 0.001	0.74
iNDF	0.48	0.44	0.43	0.39	0.021	0.036	0.17	0.58
dOM	1.35	2.18	1.96	1.80	0.100	< 0.001	0.032	0.84
TDN	1.38	2.26	2.07	1.88	0.112	< 0.001	0.043	0.97
	*g/kg*							
CP/dOM	254	265	282	301	11.4	0.062	0.054	0.94

^1^DM = dry matter; DMF = forage dry matter; DMS: supplement dry matter; DMM: milk dry matter; OM = organic matter; CP = crude protein; EE = ether extract; apNDF = neutral detergent fiber corrected for ash and protein; NFC = non-fibrous carbohydrate; iNDF = indigestible neutral detergent fiber; dOM: digestible organic matter; TDN: total digestible nutrients; CP/dOM: crude protein to digestible organic matter ratio. ^2^The calves were not supplemented (CONTROL) or supplemented without (SUP0) or with 48.4% (SUP48) or 96.8% (SUP96) inclusion of dried distillers grains. ^3^SEM: Standard error of the mean. ^4^CONTROL × SUP, contrast between supplemented and non-supplemented calves. L and Q = linear and quadratic effects of treatments. Significative when *P* < 0.10

Supplementation increased total DM, OM, CP (*P* < 0.001), EE (*P* = 0.036), and the crude protein/digestible organic matter (CP/dOM) ratio (*P* = 0.062) intake. On the other hand, IFDMI was higher for CONTROL animals (*P* = 0.051). There was no effect of supplementation on apNDF intake (*P* = 0.22) (Table 4). Supplementation linearly increased (*P* < 0.001) NFC intake. However, a decrease in NFC (*P* < 0.001) and iNDF intake (*P* = 0.036) was observed with the increasing inclusion of DDG in the supplement.

SUP calves had greater dOM and TDN intake (*P* < 0.001). However, among supplemented animals, a negative linear effect was observed for dOM (*P* = 0.032) and TDN (*P* = 0.043) with the inclusion of DDG in the supplement. Also, SUP increased digestibility of DM, OM, CP, NFC, TDN, and dOM (*P* < 0.10). However, among SUP animals, a reduction in digestibility of DM, OM, CP, NFC, and dOM was observed with the inclusion of DDG in the supplement (Table 5).

**Table 5:** Digestibility of beef calves that received different supplements during suckling phase in a tropical creep-feeding system

Item^1^	Treatment^2^				SEM^3^	*P*-value^4^		
	CONTROL	SUP0	SUP48	SUP96		CONTROL × SUP	L	Q
	*g/g*							
DM	0.557	0.675	0.634	0.614	0.016	0.002	0.035	0.60
OM	0.603	0.707	0.673	0.652	0.018	0.007	0.067	0.78
CP	0.634	0.737	0.688	0.672	0.023	0.046	0.094	0.58
EE	0.820	0.854	0.861	0.846	0.016	0.107	0.73	0.60
apNDF	0.407	0.437	0.443	0.462	0.053	0.53	0.74	0.92
NFC	0.437	0.799	0.745	0.656	0.029	< 0.001	0.010	0.64
	*g/kg MS*							
dOM	556	656	625	607	16.1	0.004	0.067	0.74
TDN	570	682	660	634	22.0	0.008	0.15	0.93

^1^DM = dry matter; OM = organic matter; CP = crude protein; EE = ether extract; apNDF = neutral detergent fiber corrected for ash and protein; NFC = non fibrous carbohydrate; dOM: digestible organic matter; TDN: total digestible nutrients. ^2^The calves were not supplemented (CONTROL) or supplemented without (SUP0) or with 48.4% (SUP48) or 96.8% (SUP96) inclusion of dried distillers grains. ^3^SEM: Standard error of the mean. ^4^CONTROL × SUP, contrast between supplemented and non-supplemented calves. L and Q = linear and quadratic effects of treatments. Significative when *P* < 0.10

Calves SUP had higher blood concentrations of IGF-1 (*P* = 0.010), glucose (*P* = 0.048), and SUN (*P* < 0.001). Albumin showed a quadratic effect (*P* = 0.061), and Globulins showed a positive linear effect (*P* = 0.067) with increasing DDG inclusion in the supplement (Table 6).

**Table 6:** Metabolic profile of beef calves that received different supplements during suckling phase in a tropical creep-feeding system

Item^1^	Treatment^2^				SEM^3^	*P*-value^4^		
	CONTROL	SUP0	SUP48	SUP96		CONTROL × SUP	L	Q
TP, g/dL	5.43	5.36	5.63	5.69	0.146	0.45	0.15	0.57
Albumin, g/dL	2.92	2.91	3.11	2.98	0.063	0.28	0.48	0.061
Globulins, g/dL	2.48	2.46	2.50	2.74	0.092	0.45	0.067	0.37
Glucose, mg/dL	78.4	83.9	86.3	83.3	2.261	0.048	0.86	0.36
IGF-1, ng/mL	275	363	377	339	21.99	0.010	0.46	0.35
SUN, mg/dL	7.99	12.2	12.8	11.1	0.667	< 0.001	0.25	0.18

^1^Total proteins (TP), Insulin-like Growth Factor 1 (IGF-1) and Serum urea nitrogen (SUN). ^2^The calves were not supplemented (CONTROL) or supplemented without (SUP0) or with 48.4% (SUP48) or 96.8% (SUP96) inclusion of dried distillers grains. ^3^SEM: Standard error of the mean. ^4^CONTROL × SUP, contrast between supplemented and non-supplemented calves. L and Q = linear and quadratic effects of treatments. Significative when *P* < 0.10

Supplementation increased (*P* < 0.001) weaning body weight (WBW), ADG, REA, and LSFT of calves (*P* = 0.075). A quadratic effect of supplementation was observed for RSFT (*P* = 0.014), but not for other performance variables related to calves (WBW, ADG, REA, LSFT) (Table 7). The BCS of the cows was not influenced by treatments (*P* > 0.10) (Table 7).

**Table 7:** Productive performance of beef calves that received different supplements during suckling phase in a tropical creep-feeding system

Item^1^	Treatment^2^				SEM^3^	*P*-value^4^		
	CONTROL	SUP0	SUP48	SUP96		CONTROL × SUP	L	Q
BCS	5.20	5.38	5.30	5.26	0.172	0.59	0.63	0.93
WBW, kg	223	251	246	242	3.985	< 0.001	0.17	0.83
ADG, kg	0.75	0.99	0.94	0.91	0.034	< 0.001	0.18	0.83
REA, cm²	39.8	44.2	46.4	46.1	1.010	< 0.001	0.24	0.35
RSFT, mm	2.28	2.41	2.92	2.31	0.144	0.14	0.64	0.014
LSFT, mm	2.83	3.57	3.60	3.05	0.243	0.075	0.18	0.36

¹BCS: final dams body condition score; WBW: calf weaning weight; ADG: average daily gain of calves; REA: calf ribeye area; RSFT: calf subcutaneous fat thickness at the rump; LSFT: calf loin subcutaneous fat thickness. ^2^The calves were not supplemented (CONTROL) or supplemented without (SUP0) or with 48.4% (SUP48) or 96.8% (SUP96) inclusion of dried distillers grains. ^3^SEM: standard error of the mean. ^4^CONTROL × SUP: contrast between supplemented and non-supplemented calves. L and Q: linear and quadratic effects of treatments. Significant when *P* < 0.10

Considering the average supplement intake and the additional gain observed, 5.26 kg of supplement were required per kg of gain. Based on these values, the partial economic analysis (Table 8) indicated higher returns under scenarios of higher calf prices and lower supplement costs.

**Table 8:** Partial net return considering feed supplement cost per kg of additional gain under different weaned calf price scenarios

Supplement cost (*R*$/kg)^1^	Calve price (*R*$/kg)					
	*R*$ 10.00	*R*$ 11.00	*R*$ 12.00	*R*$ 13.00	*R*$ 14.00	*R*$ 15.00
R$ 1.00	R$ 4.74	R$ 5.74	R$ 6.74	R$ 7.74	R$ 8.74	R$ 9.74
R$ 1.25	R$ 3.43	R$ 4.43	R$ 5.43	R$ 6.43	R$ 7.43	R$ 8.43
R$ 1.50	R$ 2.11	R$ 3.11	R$ 4.11	R$ 5.11	R$ 6.11	R$ 7.11
R$ 1.75	R$ 0.80	R$ 1.80	R$ 2.80	R$ 3.80	R$ 4.80	R$ 5.80
R$ 2.00	-R$ 0.52	R$ 0.48	R$ 1.48	R$ 2.48	R$ 3.48	R$ 4.48
R$ 2.25	-R$ 1.84	-R$ 0.83	R$ 0.17	R$ 1.17	R$ 2.17	R$ 3.17
R$ 2.50	-R$ 3.15	-R$ 2.15	-R$ 1.15	-R$ 0.15	R$ 0.85	R$ 1.85

^1^Values expressed in Brazilian Real (R$). Exchange rate variations may affect the economic interpretation of the results. However, economic outcomes are primarily driven by the relationship between feed cost and calf prices. Note: According to the study data, 5.26 kg of supplement were required per kg of additional gain. Average supplement intake: 1.053 kg/day. Additional daily gain: 0.2 kg/day

## Discussion

The average availability of pdDM was 117 g/kg BW, a value above the recommended by Paulino et al. (2008), demonstrating that forage availability was not limiting during the study, allowing animals to exercise selective grazing. Additionally, the average content of 75.7 g CP/kg DM was close to the minimum recommended value of 80 g CP/kg DM for ruminal microorganisms to optimize the use of low-quality fiber (Lazzarini et al. 2009; Detmann et al. 2010). Under these circumstances, supplementation increased nutrient supply, mainly rumen protein, optimizing forage utilization and the availability of metabolizable nutrients for continuous animal growth.

Supplemented calves exhibited greater total DM intake but reduced forage intake, consistent with a substitution effect driven by concentrate intake (Detmann et al. 2014; Lopes et al. 2017). However, increasing DDG inclusion linearly reduced total DM and OM, primarily driven by marked decline in NFC intake, reflecting the low starch content of distillers grains. This reduction in readily fermentable carbohydrates likely limited energy intake and contributed to the observed decreases in digestible organic matter (dOM) and total digestible nutrients (TDN). Although total NDF intake remained relatively unchanged, shifts in fiber fractions, including a tendency for lower iNDF intake, suggest changes in forage intake patterns, with SUP0 animals likely consuming more pasture. Despite these changes, NDF digestibility was not affected, indicating that nitrogen supply from forage remained sufficient to sustain fibrolytic activity. This is further supported by the CP/dOM ratio, which remained above the thresholds associated with ruminal conditions adequate for maximum voluntary pasture intake (Reis et al. 2016; Detmann et al. 2024). Milk intake, did not differ between SUP and CONTROL calves, indicating that supplementation did not affect suckling behavior or displace milk intake. Consistently, no changes were observed in milk production, suggesting that concentrate supplementation did not interfere with maternal milk yield or calf milk intake patterns (Lopes et al. 2016).

Increasing DDG inclusion reduced NFC intake due to its low starch content, a consequence of ethanol production processes (Adams et al. 2022). Although total NDF intake remained similar, differences in fiber fractions suggest shifts in intake patterns, with control calves consuming more forage-derived indigestible fiber. Distillers grains are characterized by high NDF content with relatively low lignin concentration, which contributes to a greater proportion of potentially digestible fiber (Schingoethe 2006).

The higher digestibility of DM, OM, CP, and NFC in SUP calves was attributed to the higher concentration of readily fermentable and digestible compounds from the grains (Almeida et al. 2018), which increased the nutritional value of the diet and, consequently, the intake of digestible nutrients, reflecting in higher TDN and dOM values.

The linear reduction in the digestibility of DM, CP, an NFC with increasing DDG inclusion likely reflects lower availability of rapidly fermentable carbohydrates and greater proportion of rumen undegradable protein (RUP), which may reduce synchronization between nitrogen and energy supply and limit microbial protein synthesis. However, reduced apparent CP digestibility does not necessarily imply lower protein supply to the animal, as RUP can contribute substantially to metabolizable protein (MP) (Putri et al. 2021; Aloba et al. 2022). Thus, metabolizable protein provides a more accurate representation of amino acid supply than apparent digestibility in diets with higher RUP inclusion (Nasem 2021). In this context, microbial protein synthesis may have contributed more substantially to MP supply in SUP0, whereas RUP likely played a greater role in SUP48 and SUP96 (Galyean and Tedeschi 2023). These changes in nutrient portioning between ruminal and post-ruminal digestion help explain the absence of negative effects on performance despite reductions in digestibility. Taken together, the results indicate that DDG inclusion alters nutrient partitioning between the rumen and intestine. This shift likely increases reliance on intestinal digestion and metabolizable protein supply, helping to maintain nutrient availability for growth. These findings reinforce the importance of evaluating diets based on metabolizable protein rather than solely on apparent digestibility (Yousefinejad et al. 2021).

Higher energy intake in supplemented calves was associated with increased circulating glucose, which likely contributed to the elevated IGF-1 concentrations observed. IGF-1, in turn, is an endocrine regulator of muscle growth in cattle, acting on glucose and amino acid metabolism and being strongly associated with nutritional status (Santos 2014). This endocrine response likely contributed to the greater performance observed in supplemented animals.

Furthermore, serum urea nitrogen (SUN) concentrations were higher in calves from the SUP group than in the CONTROL group, indicating increased nitrogen intake but also suggesting suboptimal synchronization between rumen-degradable protein and fermentable energy, which may have limited microbial nitrogen incorporation and increased ammonia conversion to urea (Cantalapiedra-Hijar et al. 2020). The lack of response to DDG inclusion indicates that this protein source did not improve nitrogen use efficiency, maintaining similar patterns of protein metabolism across treatments. Notably, SUN is sensitive to nitrogen intake (Batista et al. 2016. While some studies report improved performance at approximately 19% CP (Lopes et al. 2014), others found no differences across a wider range (15–30% CP), with greater nitrogen use efficiency at lower protein levels (Manso et al. 2025). Thus, although the CP level used in the present study (22.4%) falls within the range associated with maximal performance under tropical conditions (Carvalho et al. 2019) the elevated SUN concentrations suggest that nitrogen supply may have exceeded the animals’ capacity for efficient utilization.

SUP calves performed better compared to CONTROL. On average, supplementation provided an additional 200 g/day of weight gain, resulting in 23.3 kg more body weight at weaning. As demonstrated by our results, supplementation for suckling calves improves nutrient intake and digestibility. Consequently, it also increases the supply of metabolizable protein and energy, allowing greater deposition of muscle and adipose tissue by the animal, directly impacting performance (Carvalho et al. 2019; Lopes et al. 2017; Valente et al. 2012, 2014). Thus, in our study, SUP calves also showed greater REA and LSFT.

The partial economic analysis indicates that the viability of supplementation is highly sensitive to market conditions, particularly the relationship between supplement cost and calf value. Under the conditions evaluated, economic outcomes were more favorable when supplement costs were lower and calf prices were higher, whereas increases in supplement cost reduced profitability and, in some cases, resulted in negative returns.

This pattern can be illustrated by comparing different supplement sources. Considering a cost of R$ 2.00/kg for a traditional corn and soybean meal-based supplement, the estimated return at a calf price of R$ 11.00/kg was R$ 0.48 per kg of additional gain. In contrast, when DDG was used at R$ 1.75/kg, the return increased to R$ 1.80 per kg of additional gain, highlighting the direct impact of supplement cost on economic returns and the potential of DDG as a lower-cost alternative.

These findings indicate that, although supplementation improved animal performance, its adoption in cow–calf systems should be evaluated in light of the economic context, input availability, and price volatility, particularly in tropical production systems. It is important to emphasize that this represents a partial economic assessment, as only supplement costs were considered.

Supplementation improves intake, nutrient digestibility, and metabolism of suckling beef calves under tropical conditions, leading to better performance during the cow-calf phase and higher weaning weight. Additionally, DDG can be used as an alternative feed ingredient to replace traditional ingredients such as corn and soybean meal, without negatively affecting weight gain of suckling beef calves on tropical pastures.

## Data Availability

The datasets generated and/or analyzed during the current study are available from the manuscript and also from the corresponding author upon reasonable request.
